# Diet, Microbiota and Gut-Lung Connection

**DOI:** 10.3389/fmicb.2018.02147

**Published:** 2018-09-19

**Authors:** Swadha Anand, Sharmila S. Mande

**Affiliations:** Bio-Sciences R&D Division, TCS Research, Tata Consultancy Services Ltd., Pune, India

**Keywords:** gut-lung axis, microbiome, lung immunity, gut microbiota, lung microbiota, diet, short chain fatty acids, SCFA

## Abstract

The gut microbial community (Gut microbiota) is known to impact metabolic functions as well as immune responses in our body. Diet plays an important role in determining the composition of the gut microbiota. Gut microbes help in assimilating dietary nutrients which are indigestible by humans. The metabolites produced by them not only modulate gastro-intestinal immunity, but also impact distal organs like lung and brain. Micro-aspiration of gut bacteria or movement of sensitized immune cells through lymph or bloodstream can also influence immune response of other organs. Dysbiosis in gut microbiota has been implicated in several lung diseases, including allergy, asthma and cystic fibrosis. The bi-directional cross-talk between gut and lung (termed as Gut-Lung axis) is best exemplified by intestinal disturbances observed in lung diseases. Some of the existing probiotics show beneficial effects on lung health. A deeper understanding of the gut microbiome which comprises of all the genetic material within the gut microbiota and its role in respiratory disorders is likely to help in designing appropriate probiotic cocktails for therapeutic applications.

## Introduction

**Figure 1:**
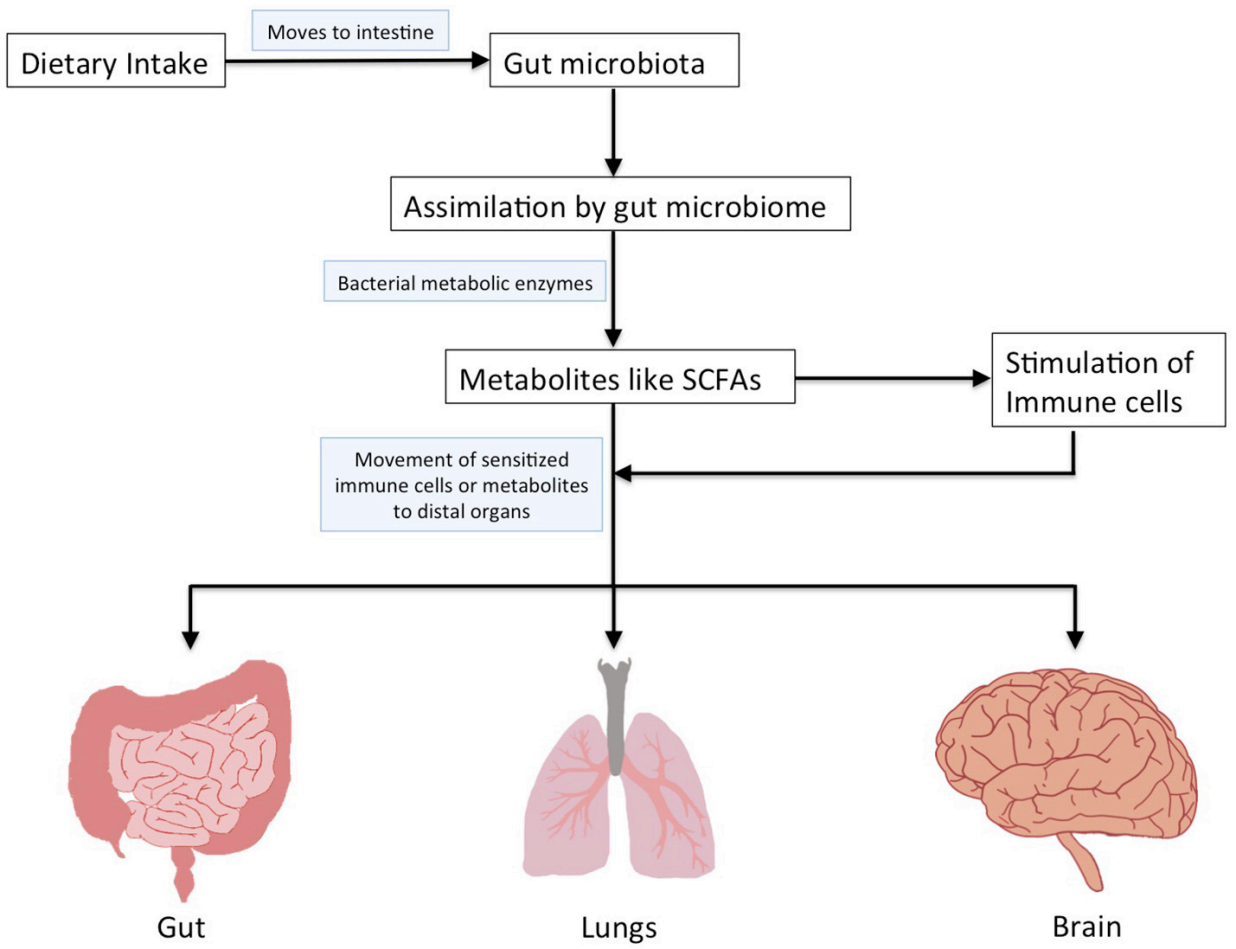
Schematic representation to depict assimilation of dietary nutrients by gut microbiome and their impact on distal organs.

The human microbiota is constituted by the microbial communities residing in various body sites (gut, skin, mouth, airways, and vagina; Cho and Blaser, 2012). Majority of these microbes are present in the gastrointestinal tract (gut) and are important in maintaining our health (Shreiner et al., 2015). Dysbiosis within this homeostasis in the gut microbiota is associated with a multitude of health conditions affecting not only gut, but also distal organs like mouth, lung, brain, liver, vagina, etc., (Shreiner et al., 2015). The gut microbial community possesses enzymatic machinery for assimilating a variety of dietary nutrients leading to release of metabolites having multiple functions in the host (Albenberg and Wu, 2014; Figure 1). It is important to understand the influence of gut microbiota and metabolites produced by them on the functioning of various organs within the body (Figure 1). The gut microbiota affects other organs either by aspiration or produces metabolites that bring about changes when they reach other organs (Marsland et al., 2015; Bingula et al., 2017).

Scientific findings indicate a link between gut microbiota and lung immunity (Marsland et al., 2015; Bingula et al., 2017). In this review, we focus on the recent developments related to influence of diet on this “Gut-Lung axis” as well as discuss the impact of different types of diet on gut microbiota and how they influence lung immunity as well as lung diseases.

## Diet and gut microbiota

A nutrient-rich diet augmented with dietary fibers is associated with a more diversified gut microbiota which is beneficial for the host (Simpson and Campbell, 2015; Xu and Knight, 2015). The metabolites produced by gut microbiome during assimilation of food significantly influence human health (Russell et al., 2013b; Joyce and Gahan, 2014; Sharon et al., 2014; Wu, 2016). The association between dysbiosis in gut microbiota and malnutrition (Gupta et al., 2011; Smith et al., 2013; Ghosh et al., 2014; De Clercq et al., 2016; Million et al., 2017) further demonstrates a link between diet and microbiota. Apart from intestinal disorders (diabetes, obesity, colon cancer, inflammatory bowel disease, etc.), changes in dietary patterns and their effects on gut microbiota are implicated in disorders of other organs like lung (asthma, COPD, etc.; Fujimura and Lynch, 2015; Panzer and Lynch, 2015) and brain (Alzheimers, depression, etc.; Dash et al., 2015; Köhler et al., 2016; Pistollato et al., 2016). The influence of dietary components on gut microbiota and associated physiological changes has been discussed in Table 1.

**Table 1:** Influence of various types of dietary components on gut microbiome and associated physiological changes.

**Dietary component**	**Effect on gut bacteria**	**Metabolic changes by gut bacteria**	**Effects on health (Lung diseases which are associated with dietary intake have been highlighted in bold-italic font)**	**References (PMIDs)**
**PROTEINS**				
Plant proteins (Whey and pea protein)	Increased *Lactobacillus* and *Bifidobacteria* Reduced pathogenic *Bacteroides fragilis* and *Clostridium perfringens*	Increased SCFA levels	Anti-inflammatory Maintenance of mucosal barrier Increased Tregs	29311977 26463271 21276631
Animal proteins	Increased *Bacteroides* and *Alistipes*	Reduced SCFA levels	Increased inflammatory disorders High risk of Inflammatory Bowel Disease (IBD) Increased levels of proatherogenictrimethylamine-N-oxide (TMAO) increases risk of cardiovascular disease (CVD)	29311977 29534465 20679230 22055893 28388917
High protein/carbohydrate ratio	Reduced *Roseburia* and *Eubacterium*	Less fecal butyrate	Increased inflammation	21389180 29534465
**CARBOHYDRATES**				
Digestible carbohydrates (lactose)	Increased *Bifidobacteria* Lactose leads to high *Lactobacillus* Lactose high SCFA production	Increased SCFA levels	Anti-inflammatory	22435727 28388917 22435727
Non-digestible carbohydrates (Wheat bran)	Increased *Bifidobacteria* and *Lactobacillus*	Increased SCFA levels	Increased inflammatory disorders	26636660 29416529
Non-digestible carbohydrates: Dietary Fiber (DF), Resistant starch (RS) and whole grain barley	Increased *Roseburia, Ruminococcus* and *Eubacterium*	Increased fecal butyrate	Reduced inflammation **DF shows beneficial effects in Asthma, Cystic fibrosis, COPD**	29042495 24440361 18063592
Non-digestible carbohydrates Fructooligosaccharides (FOS), Galactooligosaccharides (GOS) and polydextrose	Increased *Roseburia*, and *Eubacterium* Reduced *Enterococcus* and *Clostridia*	Increased fecal butyrate	Reduced inflammatory response **GOS alleviated asthma and eosinophillia in murine models**	23951074 25849971 27523186
**FATS**				
Saturated fat (lard fat)	Decreased *Bifidobacteria* and *Eubacterium* species	Reduced SCFA levels	Increased inflammatory disorders ***Asthma with high fat low carbohydrate diet*** Control metabolic endotoxemia-induced inflammation TLR activation, Adipose tissue inflammation (obesity), insulin resistance (Diabetes) Increases risk of cardiovascular disease	26321659 28804483 28388917
Unsaturated fat (fish oil)	Increased *Roseburia, Ruminococcus* and *Eubacterium*	Increased fecal butyrate	Reduced inflammation **Omega-3 fatty acids ameliorate asthma, pneumonia and COPD**	28388917 26784651 28951525 29215589
**SPECIFIC DIET REGIMES**				
Western: High in animal protein and saturated and trans fat, Low in fiber	Decreased *Bifidobacteria* and *Eubacterium* species	Reduced SCFA levels	Increased inflammatory disorders **Studies on asthma patients show mixed outcomes**	29276171 26011307 28388917
Gluten free	Increased *Roseburia, Ruminococcus* and *Eubacterium*	Increased fecal butyrate	Reduced inflammation	27102333 25651995
Mediterranean: Beneficial mono-unsaturated and poly-unsaturated fatty acids, high levels of polyphenols and other antioxidants, high fiber and low glycemic carbohydrates, more vegetables less animal protein	Increases in *Lactobacillus, Bifidobacterium*, and *Prevotella* Decreases in *Clostridium*	Higher SCFA levels	Reduced inflammatory response Less adherence to Mediterranean diet associated with increase in TMAO and therefore, CVD Less adherence to Mediterranean diet associated diabetes and obesity	28789729 24987952 26416813

The role of diet in determining gut microbiota has been studied in different stages of life. Although the colonization of microbes in the gut begins at birth, their configuration keeps changing until a stable state is attained which is closer to the adult microbiota (Bergström et al., 2014; Backhed et al., 2015; Shukla et al., 2017). The composition of gut microbiota in early stages is determined by various factors, including mode of delivery and feeding, antibiotic exposure and surrounding environment. For example, the taxonomic composition of gut microbiota in breast-fed babies (higher *Bifidobacteria, Lactobacilli, Staphylococci*, and *Streptococci*) considerably differs from infants who are formula-fed (higher *Bacteroides, Clostridia*, and *Proteobacteria*; Timmerman et al., 2017). The gut microbiota in infants becomes stable after solid food diet is introduced, after which the composition is largely determined by dietary intake (Timmerman et al., 2017).

The gut microbiota of younger adults highly depends on their diet and lifestyle (Turnbaugh et al., 2009). As compared to individuals consuming Agrarian diet (low fat, higher plant products), those on Western diet (high fat and sugars) show significant decrease in *Bacteroidetes* (De Filippo et al., 2010). Children from rural Africa (having diet high in plant polysaccharide/protein and fiber; less animal protein) have higher levels of *Actinobacteria* and *Bacteroidetes* as against *Firmicutes* and *Proteobacteria* which occur in higher numbers in Western European children (diet protein, sugar, fat and low on fiber; De Filippo et al., 2010). The amount of short chain fatty acids (SCFAs) is about four times higher in African population with higher abundances of certain bacteria (*Xylanibacter, Prevotella, Butyrivibrio*, and *Treponema*) that are capable of digesting plant polysaccharides to yield SCFAs (De Filippo et al., 2010). The increased SCFA production by gut microbiome leads to lowering of pH in their gut, thereby reducing the pathogenic species like *Escherichia coli* and *Enterobacteriaceae* (Zimmer et al., 2012; Glick-Bauer and Yeh, 2014).

The later stages of life have also been associated with changes in gut microbiota and might be attributed to factors like lifestyle, diet, immune strength, altered gut morphology and physiology, frequent infections, hospitalizations, medication, etc., (Vemuri et al., 2018). The diversity of gut microbiota reduces with aging, but the associated changes may vary across different geographies. Although it is unclear whether these changes are a cause or an effect of aging, the maintenance of gut homeostasis might be crucial for longevity. Apart from the taxonomic changes, aging has been associated with a reduced metabolic potential of microbiome, including less production of SCFAs (Kumar et al., 2016; Nagpal et al., 2018). These changes could be related to changes in dietary pattern with age. It has been speculated that the metabolites produced by gut microbiota might have an effect on longevity (Kumar et al., 2016; Nagpal et al., 2018).

The above-mentioned studies establish that differences in diet play an important role in determining the composition of the gut microbiota. The physiological impact of changes in gut microbiota with variation in dietary patterns is governed by differences in microbial metabolome profiles. *In vitro* and *in vivo* studies have shown that food components are utilized by gut bacteria to biosynthesize metabolites (like SCFAs from soluble fibers), which modulate the host immune system (Kosiewicz et al., 2011; Maslowski and Mackay, 2011; Tilg and Moschen, 2015; Tomkovich and Jobin, 2016). Thus, the influence of diet on intestinal microbial dynamics suggests “Diet-Microbiota-Immunity” link and delineates their role in maintenance of health (Kosiewicz et al., 2011; Maslowski and Mackay, 2011; Tilg and Moschen, 2015; Tomkovich and Jobin, 2016).

## Roles of short chain fatty acids (SCFAs) produced by gut microbiota

SCFAs (acetate, propionate, and butyrate) promote recruitment as well as maturation of immune cells which provide protection against inflammatory response (Vinolo et al., 2011). They influence host metabolic activities by serving as a link between dietary fiber, commensal microbes and host (Ha et al., 2014; Kasubuchi et al., 2015; Yang and Duan, 2018). SCFAs produced in the gut are distributed systemically and are either utilized to provide energy or as signaling molecules (Morrison and Preston, 2016).

### Energy and metabolism

A large part of SCFAs produced in the gut are utilized as energy source and can provide as high as ~10% of our daily caloric requirement (Bergman, 1990). Such roles of SCFAs thus indicate their association with metabolic disorders like obesity and diabetes (Cho et al., 2013; Chen et al., 2014; Ríos-Covián et al., 2016). *In vivo* and *in vitro* studies in mice have further shown that butyrate and propionate might induce hormone production by gut bacteria (Lin et al., 2012; Tolhurst et al., 2012). The SCFAs bind to G-protein coupled receptors (GPCRs; Den Besten et al., 2013; Sun et al., 2017) which sense metabolites and trigger signaling pathways leading to anti-inflammatory effects on the host immune responses (Sun et al., 2017).

### Integrity of gut epithelium

The SCFAs in gut decrease the luminal pH, thereby curtailing growth of pathogenic microorganisms. Acetate production by *Bifidobacteria* has been observed to inhibit growth of enteropathogens in mice (Fukuda et al., 2011, 2012). Moreover, *in vitro* and *in vivo* studies have indicated that higher butyrate levels lead to increased mucin production which reduces bacterial adhesion and improves epithelial integrity (Jung et al., 2015; Jiminez et al., 2017).

### Systemic immunity

Studies on mice have shown that butyrate production leads to an increase in the number of Treg cells. *In vitro* studies further established the role of butyrate in regulation of forkhead transcription factor FOXP3 (a lineage specification factor). Butyrate works as an inhibitor of histone deacetylases (HDAC) and promotes acetylation of histone H3 at the promoter of the gene encoding FOXP3 (Zeng and Chi, 2015; Kespohl et al., 2017; Supplementary Figure S1). SCFAs also influence the development of dendritic cells and inflammatory cytokines, thereby regulating the intestinal macrophages (Chauvistré et al., 2014; Supplementary Figure S1). Multiple roles of SCFAs in energy and metabolism support activation/differentiation of B cells as well as production of antibodies (IgM/IgA; Supplementary Figure S1; Kim, 2016; Kim et al., 2016).

### Gut-lung axis

Dysbiosis in gut microbiota is associated with lung disorders and respiratory infections (Trompette et al., 2014; Shukla et al., 2017). For example, reduction in genus *Bifidobacteria* and increase in *Clostridia* in the intestine are associated with asthma in early life (Kalliomaki et al., 2001). Furthermore, murine studies show that depletion of certain species within the gut microbiota due to antibiotic intake influences lung diseases and allergic inflammation (Russell et al., 2013a; Dharmage et al., 2015; Metsälä et al., 2015). For instance, studies on mouse models indicate that removal of sensitive gut bacteria after administration of neomycin leads to an increase in the susceptibility to influenza virus infection in lungs (Ichinohe et al., 2011; Looft and Allen, 2012).

The changes in lung microbial community also influence the composition of gut microbiota. For example, influenza virus infection in the respiratory tract (in mice models) increases *Enterobacteriaceae* as well reduces *Lactobacilli* and *Lactococci* in the intestinal microbiota (Looft and Allen, 2012). The dysbiosis in lung microbiota upon administration of Lipopolysaccharide (LPS) in mice is accompanied by disturbances in their gut microbiota due to movement of bacteria from their lung into the bloodstream (Sze et al., 2014).

All the above-mentioned findings corroborate that gut and lung are intricately linked organs which influence each others' homeostasis.

### Gut microbiota influence lung immune response

Perturbation of the normal gut microbiota may be associated with development of experimental asthma and other respiratory disorders, as demonstrated in animal models (Noverr et al., 2004). Evidence is increasing in support of a “common mucosal response,” which states that the effects of gut microbiota on the mucosal immunity may have an influence on the immune responses at distal mucosal sites, including that in lung (Mestecky, 1987; McGhee and Fujihashi, 2012; Hauptmann and Schaible, 2016; Date et al., 2017; Ipci et al., 2017). Further, gut bacterial cells as well as metabolites biosynthesized by them might stimulate the immune response in distal sites (Hauptmann and Schaible, 2016).

#### Migration of immune cells from gut to lung by common mucosal immune system (MIS)

The Mucosal Immune System (MIS) constitutes inductive as well as effector sites which are defined on the basis of their functional properties and anatomy. The immune cells migrate from these mucosal inductive to effector sites through the lymphatic system (McGhee and Fujihashi, 2012). This transfer of cells determines the immune response in different organs (gastro-intestinal tract, lung, etc.,). Mucosal inductive sites form mucosa-associated lymphoid tissue (MALT) that comprises of gut-associated lymphoid tissues (GALT) and nasopharyngeal-associated lymphoid tissues (NALT; McGhee and Fujihashi, 2012). The GALT consists of organized lymphoid tissues (mesenteric lymph nodes and Peyer patches) which work as inductive sites. It also possesses more diffusely scattered effector sites in the intestinal lamina and epithelium (Bekiaris et al., 2014; Brugman et al., 2015). The MALT is covered by microfold (M) cells which take up antigens present in the lumen of intestinal mucosa and transfer them to Dendritic cells (DCs) in the subepithelial regions (Zarzaur and Kudsk, 2001; Azzali, 2003; Cesta, 2006). These DCs carry antigens into inductive sites (Peyer's patch or mesenteric lymph nodes) where they bring about the initiation of mucosal T and B cell responses (McGhee and Fujihashi, 2012; Samuelson et al., 2015). The MALT possesses regions rich in T cells as well as areas populated by B cells harboring surface IgA-positive (sIgA^+^) B cells in significant numbers. Recent findings indicate that GALT-DCs can act as “antigen presenting cells” (APCs), thereby influencing differentiation and antibody secretion in B cells (Qi et al., 2006). These newly generated IgA-producing cells in inductive sites move out from GALT into the bloodstream and home to the intestinal effector site lamina propria where they act as defense against intestinal pathogens. The mucosal effector sites, including the lamina propria regions of the gastrointestinal tract, comprise of IgA-producing plasma cells and memory B and T cells. The CD4+ T helper cells (both CD4+ Th2 or CD4+ Th1 cells) support the development of IgA- producing plasma cells (McGhee and Fujihashi, 2012).

**Figure 2:**
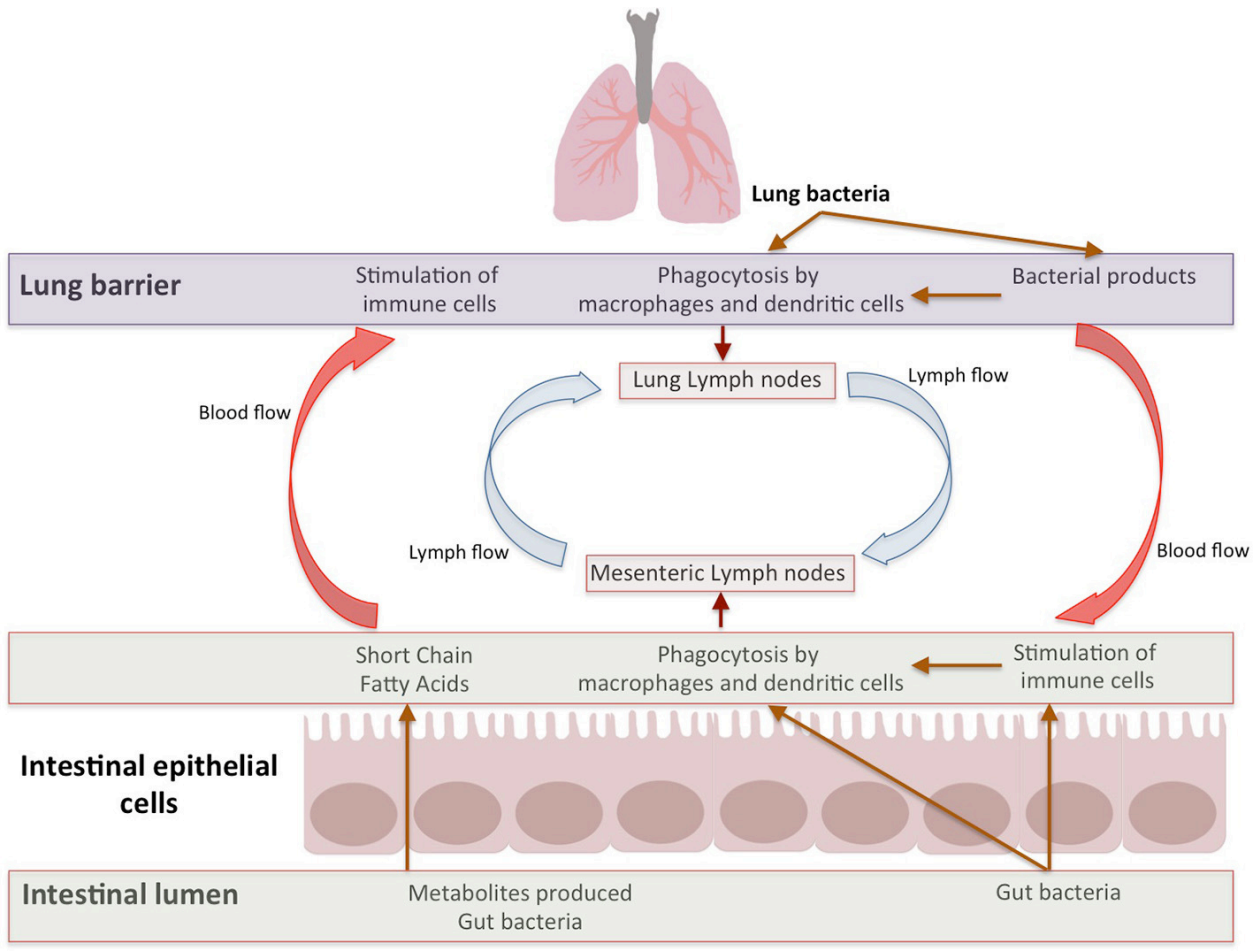
Bidirectional Gut-Lung axis. The metabolites like SCFAs produced by gut bacteria move through blood stream to stimulate immune response in lung and different factors from lung effect gut immune response. Apart from metabolites, the immune cells induced by multitude of antigens move through lymphatic duct between both these organs which leads to modulation of immune response in both organs.

After stimulation by antigens (like gut pathogens) targeting the mucosa, plasma cells produce dimeric immunoglobulin A (IgA; with specificities toward antigens in mucosal tissues) which are secreted by epithelia expressing polymeric Ig receptor (IgR; Lycke and Bemark, 2012; Rios et al., 2016). The presence of commensals in gut is critical for controlling the induction and functioning of IgA. For example, IgA positive plasma cells are in lesser numbers in neonates and germ-free (GF) animals (Macpherson et al., 2005; Harris et al., 2006). The immunity and IgA production enhances after colonization by gut bacteria. The T and B cells, induced in Peyers patches, can move into the circulation and migrate to intestinal as well as extra-intestinal sites (including bronchial epithelium and lymphoid tissues; Matsuno et al., 2010). Such B-cells produce IgA which can be transported to the mucosal surface, thereby passing “immunological information” between different organs (Roth et al., 2014; Bingula et al., 2017). Thus, the lymph and/or bloodstream act as a link between the gut (where primary sensitization occurs) and the affected site on the lung (He et al., 2017; Figure 2).

#### Gut bacterial cells or metabolites biosynthesized by them influence lung immunity

The immune cells which reside in the intestinal lamina propria and mesenteric lymph nodes can neutralize most of the translocating bacteria (Gross et al., 2015; Bingula et al., 2017). The surviving bacteria as well as the fragments of dead bacteria travel from the mesenteric lymphatic system and enter systemic circulation (Bingula et al., 2017). Further, several factors or metabolites (e.g., SCFAs) produced by gut microbiome enter systemic circulation via the intestinal lymphatic system. These bacterial fragments and metabolites can modulate lung immune response (Trompette et al., 2014; Marsland et al., 2015; Gray et al., 2017; Mcaleer and Kolls, 2018; Figure 2).

#### Homing receptors determine tissue specificity of immune cells

The immune cells (after leaving the bloodstream) destined to home into specific sites, interact with high endothelial venules (HEVs) on these effector sites (Matsuno et al., 2010). The adhesion mechanisms in mucosal effector sites involve interaction between mucosal addressin molecule MAdCAM-1 (expressed on HEVs in Peyers patches and Mesenteric Lymph nodes) and integrin α_4_β_7_ (expressed on lymphocytes; Matsuno et al., 2010; Supplementary Figure S2). The memory/effector lymphocytes further express tissue-specific chemokine receptors which contribute to their mucosal localization (Hart et al., 2010). The T-effector cells and IgA secreting plasmablasts destined to home in the small intestine show higher expression of CCR9 receptor which binds local ligand CCL25 (TECK; Matsuno et al., 2010). On the other hand, CCR10 is expressed by plasmablasts targeted toward large intestine where they interact with ligand CCL28 (MEC; Seong et al., 2017). The DCs in GALT possess capability to produce Retinoic Acid (RA) which induces the expression of these homing receptors (integrin α_4_β_7_ and CCR9) in T as well as B cells and thereby boost their migration to small intestine (Seong et al., 2017).

In contrast to the above mentioned specific interactions, certain non-tissue specific interactions allow homing of lymphocytes to multiple mucosal sites (Supplementary Figure S2). Interactions between PNad and L-selectin as well as VCAM-1 and α_4_β_1_ integrins are non-tissue specific (Sun et al., 2014). Further, the expression of certain chemokine receptors (CCR5, CXCR3, etc.) are also not tissue specific (Thomas et al., 2007). It is speculated that the immune cell homing in lungs might occur through non-tissue specific interactions (Mateer et al., 2015). However, no lung specific homing receptors are characterized till date. Further, pulmonary T-cells express these non-specific chemokine receptors.

The expression of the above-mentioned non-specific receptors is higher in the lymphocytes residing in intestinal regions during inflammation (IBD etc.; Ye et al., 2017). These gut immune cells might get into the systemic circulation and interact with lung tissues possessing ligands to bind these non-tissue specific receptors. This is likely to cause mis-homing of these lymphocytes in the lung tissues (Mateer et al., 2015). The T effector memory (TEM) cells formed during gut inflammation might use their L-selectin/CD62L to interact with PNAd ligands present on lung endothelial surface once they enter systemic circulation (Golubovskaya and Wu, 2016). Such mechanisms explain how gut lymphocytes (TEMs and IgA plasmablasts) can migrate to lung lymphoid tissues. Thus, lung and gut form part of the mucosal immune system and an inflammatory response in one of these organs may be mirrored in the other.

### Lung bacteria influence intestinal immunity

Although the above-mentioned aspects account for the effect of gut microbiota on lung immunity, the reverse has been less explored. A number of studies in different animal models have established that the manifestations of pneumonia due to multi drug resistant *Staphylococcus aureus* or *Pseudomonas aeruginosa*, which originate in the lung, is likely to induce intestinal injury (Lobo et al., 2005; Perrone et al., 2012). Pneumonia caused by *P. aeruginosa* leads to a reduction in gut epithelial proliferation and blocks the M-phase of cell cycle (Coopersmith et al., 2003; He et al., 2017). Studies have shown that the lung microbiota is formed by the micro-aspiration of microbiota from oropharynx part of the upper respiratory tract (URT) to the lung (Bassis et al., 2015; Dickson et al., 2015, 2017). In addition, studies performed on C57/Bl6 mice show that dysbiosis in the airway microbiota by intra-tracheal single dose of lipopolysaccharide leads to the movement of lung bacteria into the bloodstream. This causes an increase in the bacterial load in intestine, thereby disrupting the gut microbial community (Sze et al., 2014). This dysbiosis could be due to the interaction between translocated lung immune cells and gut microbiota. Therefore, the gut–lung axis is a bidirectional loop which is stimulated by the changes in gut or lung immunity (Bingula et al., 2017; Budden et al., 2017; Figure 2).

### Gut microbiota modulates development of allergic responses and cystic fibrosis

Low gut microbial diversity in the first month of life of an individual is correlated with the occurrence of asthma (Russell et al., 2012; Ranucci et al., 2017). The impairment of gut microbiota balance due to usage of antibiotics increases the risk of asthma (Dharmage et al., 2015; Metsälä et al., 2015). For example, microbial dysbiosis due to the use of macrolides in early life (which depletes *Actinobacteria* and increases abundances of *Bacteroidetes* and *Proteobacteria*) is correlated with an increased risk of asthma in 2–7 years old Finnish children (Korpela et al., 2016). Similarly, neonatal mice administered with vancomycin show significant changes in gut microbiota which is accompanied by an increase in allergic asthma as compared to control mice (Russell et al., 2012).

Canadian children at risk of asthma show decreased abundances of *Lachnospira, Veillonella, Faecalibacterium*, and *Rothia* in their gut (Arrieta et al., 2015). Further, inoculations of these bacteria in germ free mice show protection against airway inflammation and asthma as compared to mice which are given fecal transplantation without these bacteria (Arrieta et al., 2015). There exists an association between lower numbers of certain bacteria (*Bifidobacteria, Akkermansia* and *Faecalibacterium*) and higher risk of developing atopy/asthma in neonates (Fujimura et al., 2016). The genera *Faecalibacterium* and *Lachnospira*, which are reduced in asthmatic patients, comprise of strains that are capable of butyrate production utilizing pyruvate as substrate (Anand et al., 2016). Out of the four pathways which can produce butyrate, this butyrogenic pathway (from Pyruvate) is a marker for gut commensals (Anand et al., 2016). This reaffirms the importance of butyrate production by beneficial gut bacteria in maintaining the lung immune homeostasis.

Feces and gut lavage samples from Cystic fibrosis (CF) patients show reduced microbial diversity as well as increased intestinal inflammatory markers (Bruzzese et al., 2014). As compared to healthy children, those suffering from CF have reduced abundances of beneficial bacteria like *Eubacterium rectale, Bacteroides vulgatus, Bacteroides uniformis, Faecalibacterium prausnitzii, Bifidobacterium catenulatum*, and *Bifidobacterium adolescentis* (Bruzzese et al., 2014). *E. rectale* and *F. prausnitzii* possess capability to produce butyrate using Pyruvate as substrate (Anand et al., 2016). Further, studies on pediatric fecal samples show that CF patients possess gastrointestinal environments which favor higher SCFA catabolism. The dysbiotic microbiomes in CF children display a decreased capacity for fatty acid biosynthesis and an increased potential for degrading SCFAs (including butyrate and propionate; Manor et al., 2016). Although, administration of probiotics to CF patients shows improvement in respiratory as well as gastro-intestinal clinical outcomes, the evidence has certain limitations (Ananthan et al., 2016; Anderson et al., 2017; Nikniaz et al., 2017). Meticulously designed random controlled trials with well-assessed clinical outcomes are required to ascertain the strains/species which can be used to alleviate lung diseases as well as the dosage required for desirable outcomes.

The reduction in the abundance of bacteria utilizing Pyruvate to produce butyrate in the above-mentioned diseases (asthma and CF) is in confirmation with a similar decrease observed in patients with allergic responses. This indicates the potential of utilizing this pathway (and bacteria possessing it) in early diagnosis of respiratory diseases. Further, these bacteria can find potential applications as probiotics for treatment of several lung diseases like allergy, COPD, asthma, etc. The influence of dietary intake on butyrate production highlights a link between nutrition and lung immunity.

### Nutrition and lung function

A fiber rich diet changes not only the intestinal microbiota, but also affects the lung microbiota, indicating influence of nutrition on lung immunity (Trompette et al., 2014; Halnes et al., 2017). The dietary fiber increases SCFA levels in blood, thereby providing protection against allergic inflammation in lung (Trompette et al., 2014; Halnes et al., 2017). These findings emphasize the importance of diet and composition of gut microbiota in determining lung immune response.

Fiber rich diets show association with better lung function and lesser risk of lung disorders. A study based on 120,000 individuals who were followed up for around 12–16 years indicated that a healthy diet can lead to ~33% decline in chances of COPD (Varraso et al., 2015). The beneficial effect of fiber on lung function is clinically more significant in case of smokers, thereby indicating the potential of utilizing dietary modifications to tackle respiratory disorders. High fiber diet leads to reduction in mortality from respiratory disease (Varraso et al., 2015). A multitude of experimental interventions indicate the role of high fiber diets in modulating innate immunity, which can be corroborated by reduction in the levels of inflammatory markers (CRP and IL-6). Similarly, dietary fiber leads to a reduction in serum CRP by 20–30% from baseline (King et al., 2007).

### Can probiotics be used for treating lung diseases?

There has been a growing interest in addressing the effect of probiotics on lung disorders such as asthma and COPD. Interestingly, studies on mice models indicate that Treg cells, which down-regulate the allergic response, can be induced by the administration of probiotic bacteria like *Lactobacillus rhamnosus, Bifidobacterium lactis*, and *Bifidobacterium breve* (Feleszko et al., 2007; Jang et al., 2012; Toh et al., 2012; Sagar et al., 2014). Similarly, administration of either *Lactobacillus casei shirota* or *L. rhamnosus GG* in Cystic Fibrosis patients leads to a reduction in exacerbation symptoms (Toh et al., 2012; West et al., 2017). Further, *in vitro* administration of *L*. *rhamnosus* and *B*. *breve* suppresses the pro-inflammatory mediators induced by exposure of macrophages to cigarette smoke. These findings may indicate importance of probiotics in treatment of cigarette smoke induced diseases like COPD (Mortaz et al., 2015).

Probiotics have shown promising results in improving inflammatory conditions (e.g., IBD) as well as regulating innate immunity using toll-like receptors and the corresponding signaling pathways (Toh et al., 2012; West et al., 2017). Further, they enhance barrier function in the intestine, thereby preventing antigens like lipopolysaccharide (LPS) from leaking through. Such beneficial roles of probiotics make them potential candidates for treatment of inflammatory diseases like IBD, allergy, COPD, asthma, etc.

Probiotics also show promising results in the field of lung oncology. Oral feeding of probiotic strain *Lactobacillus acidophilus* to mice lung cancer model undergoing cisplatin treatment shows reduction in tumor size and higher survival rate (Kelly et al., 2001; Gui et al., 2015). Further, gut microbiota has the potential to control lung cancer and has been attributed to the changes in antitumor immunity (Sivan et al., 2015). For example, when a mouse melanoma model is orally administered with *Bifidobacterium* cocktail (*B. bifidum, B. longum, B. lactis*, and *B. breve*), the tumor is observed to be controlled to an equal efficacy as that seen with PDL1 specific antibody therapy. This PDL1 specific antibody therapy has shown promise in the treatment of advanced Non-small Cell Lung Cancer (NSCLC; Sivan et al., 2015). The activated T-cells express programmed death protein-1 (PD-1) receptor that interacts with tumor-expressed ligands PD-L1/L2 and leads to an inhibition of T-cell activation, thereby promoting tumor cells escaping immunity. Antibodies against PD-1/PD-L1 prevent this interaction, thereby leading to T cell activation and thus acting as an immunotherapy against tumor cells (Valecha et al., 2017). On the other hand, *Bifidobacterium* cocktail treatment leads to an up regulation of 760 genes involved in multiple aspects of immune response like CD8+ T cell activation and co-stimulation, dendritic cell maturation, antigen processing presentation and type I interferon signaling (Sivan et al., 2015). Also, feeding *Bacteroides fragilis* to germ-free mice enhances the maturation of dendritic cells due to cross-reactivity between epitopes of this bacteria and the tumor (Vétizou et al., 2015). Interestingly, an improvement in advanced lung cancer patients is observed when *Enterococcus hirae* and *Barnesiella intestine hominis* are administered in combination with chemo-immunotherapy (Daillère et al., 2016). Both these strains can be considered as potential “oncomicrobiotics” for augmenting the efficacy of cancer treatments (Daillère et al., 2016). Probiotics show potential in modulating the immune response which can prove beneficial in handling a variety of lung diseases. Although, most of the studies on effect of probiotics have been performed with regards to lung cancer, the influence of gut microbiota on other lung diseases (discussed earlier) indicates promising opportunities for their treatment as well.

Despite the beneficial effects of probiotics on the aspects of human immune response that influence allergy and inflammation, a few inconsistencies pertaining to their efficacy in clinical trials have been observed (Vliagoftis et al., 2008; Elazab et al., 2013; Zuccotti et al., 2015). The subject population in these random controlled trials included children and adults with intermittent to mild persistent asthma. The durations of the studies ranged between 4 and 14 months. The microorganisms used included strains of lactic acid bacteria (*L. casei, L. rhamnosus*, and *L. acidophilus*, etc.,) as well as other genera including *Enterococcus* and *Streptococcus*. The placebo comprised of products having the same acidity and taste, but which lacked live microorganisms. The trials showed no significant differences in several laboratory parameters (forced expiratory volume in 1 s, blood eosinophil, serum total IgE, etc.) between the probiotic treated groups and the placebo treated individuals (Wheeler et al., 1997; Giovannini et al., 2007). Further, another trial indicated no considerable differences between the intervention and the placebo groups, in terms of clinical outcomes like life quality, number of asthma episodes and usage of medicine (Wheeler et al., 1997; Giovannini et al., 2007; Stockert et al., 2007). It is to be noted that these findings are based on a few clinical studies with smaller patient cohorts. The inconsistencies in the results of these studies make it difficult to conclude whether probiotics have beneficial roles in patients with respiratory disorders. Apart from recruitment of a heterogenous population, the design of these studies involved administration of different types and quantity of probiotics for variable durations. Further, symptom-based scoring systems used to evaluate the effects of interventions were also different. Thus, it is difficult to compare such studies in order to conclusively delineate the efficacy of probiotics in treatment of respiratory diseases.

## Conclusion

The dietary intake of an individual determines the taxonomic constitution of his/her gut microbial community. The assimilation of dietary nutrients by gut microbiome leads to the production of metabolites which play significant roles in human health. Metabolites like SCFAs significantly influence the systemic immunity of not only gastro-intestinal system, but also other organs through lymphatic and circulatory systems. Thus, dysbiosis in gut microbiota affects not only gastrointestinal tract, but also influences the health of other distal organs. For example, the common mucosal system ensures that antigens introduced in gut are capable of eliciting an immune response in lung. Although the impact of changes in lung microbiota on the intestine has also been observed, an understanding of the system will require multiple mechanistic studies and clinical intervention trials. The increasing understanding of lung microbiota and its influence on the immune system might lead to more insights into these mechanisms.

The beneficial effect of pre- and probiotics have been observed in several lung ailments, although the results are inconsistent. These inconsistencies may arise because of dosage of the pre/pro-biotic administered as well as the strains used as probiotics. It is to be noted that probiotics usually comprise of a few strains, which might not be able to function in certain dysbiotic communities. Further, the efficacy of different strains might differ with changes in environmental states and geographical location. These strains might show better efficacy in the presence of other bacterial species or strains which coexist in a natural environment. Therefore, in order to delineate benefits of such treatments in lung diseases, an understanding of functional interactions within the entire microbial community is necessary. Further, understanding the metabolic potential of each strain and its capability to survive in conditions of interest also needs to be assessed. Our analyses on gut metagenomes in asthma, allergy and CF patients show decreased abundances of pyruvate utilizing butyrate-producing bacteria. This finding emphasizes the potential of utilizing the (Pyruvate -> Butyrate) pathway for diagnosis and assessment of lung diseases. In addition, our observation can be utilized for designing potential probiotic cocktails for treatment of lung disorders. Thus, the information based on the interactions amongst host genetics, diet and microbiota can be potentially used for designing personalized precision approaches for prevention as well as treatment of lung disorders.

## Author contributions

SA and SM designed the review and drafted the manuscript. Both authors approved the final version of the manuscript for submission.

### Conflict of interest statement

SM and SA are employed by company Tata Consultancy Services Ltd., (TCS) and are part of TCS Research. Both authors declare no competing interests. The funders had no role in study design, data collection and analysis, decision to publish or preparation of the manuscript.
