# Supplementary material for: Diet, Microbiota and Gut-Lung Connection
Source: Front Microbiol. 2018 Sep 19;9:2147. doi: 10.3389/fmicb.2018.02147 (PMC6156521; doi:10.3389/fmicb.2018.02147)
Supplement: Supplementary file 1 [file Image_1.PDF]

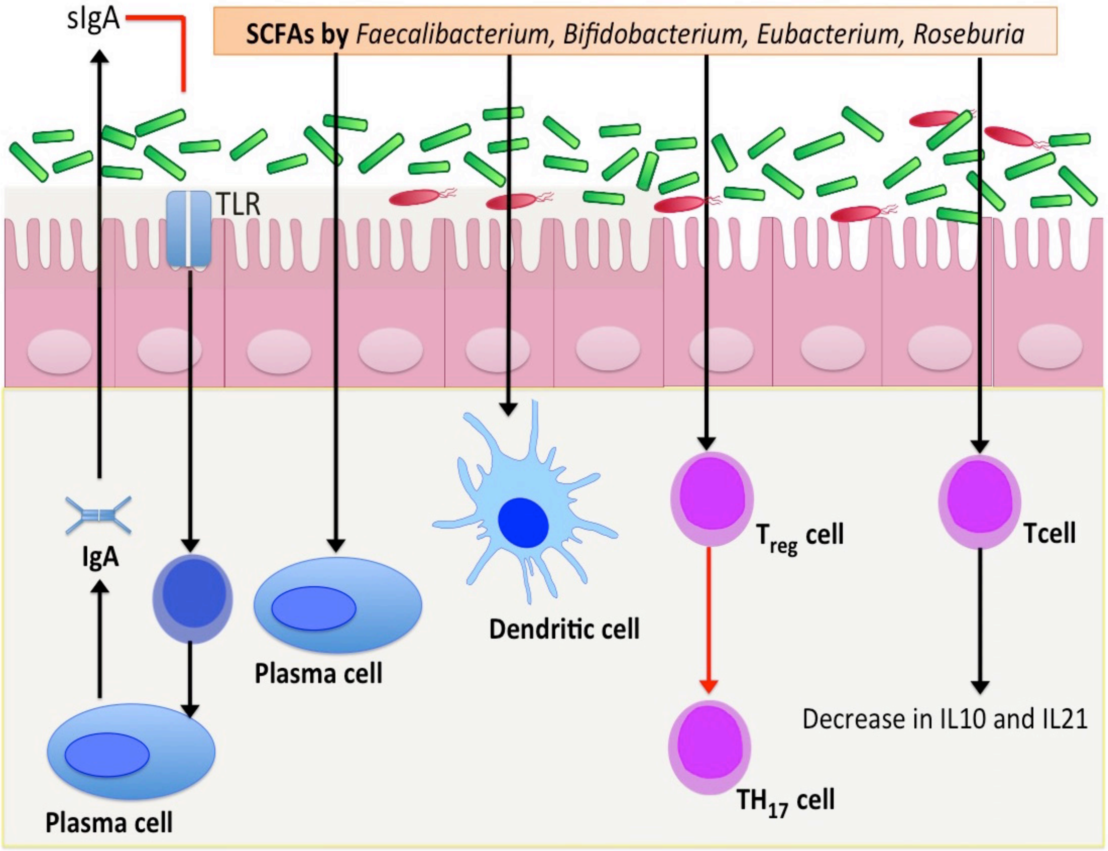

Supplementary Figure S1
Role of SCFAs produced by commensals (Faecalibacterium, Roseburia, Bifidobacterium and Eubacterium) on the immune response.
Magenta: Effect on Treg cells and cytokines leading to anti-inflammatory immune response
Blue: Effect on plasma cell to regulate differentiation and antibody IgA production
