# Supplementary material for: Diet, Microbiota and Gut-Lung Connection
Source: Front Microbiol. 2018 Sep 19;9:2147. doi: 10.3389/fmicb.2018.02147 (PMC6156521; doi:10.3389/fmicb.2018.02147)
Supplement: Supplementary file 2 [file Image_2.PDF]

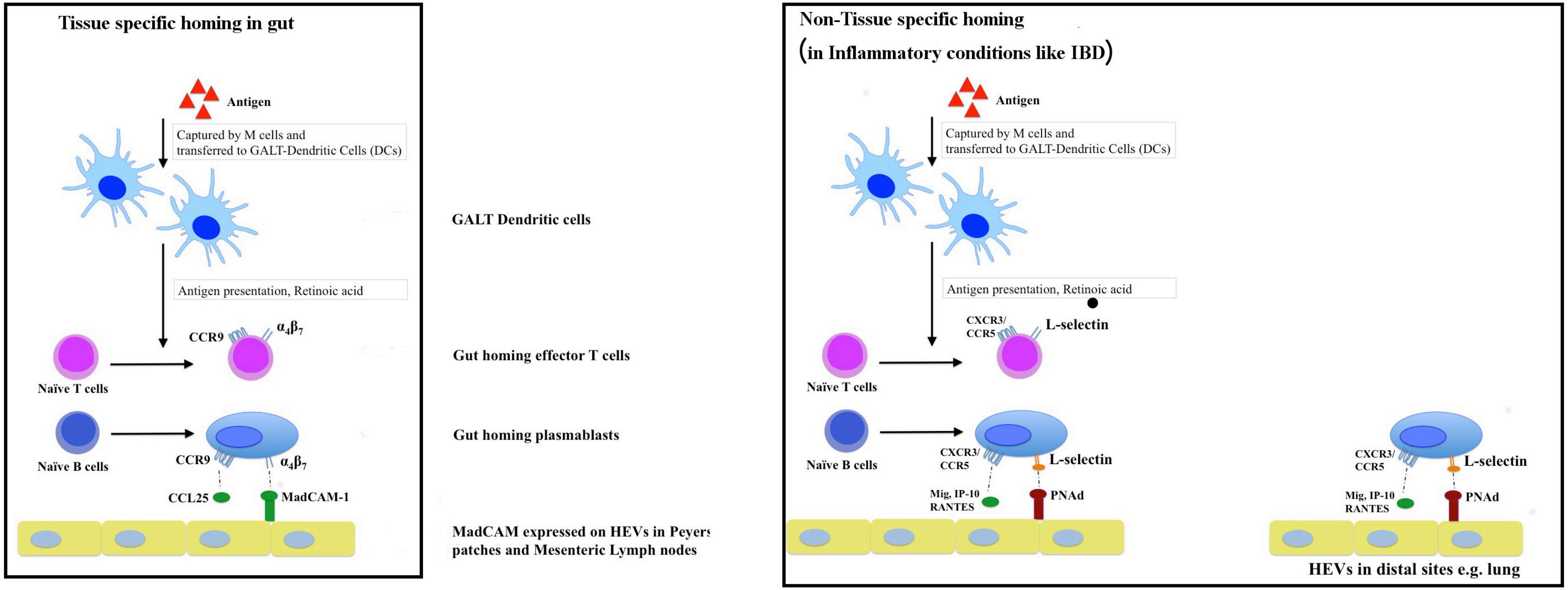

Supplementary Figure S2

The figure depicts role of tissue-specific and non tissue-specific homing receptors in immune cell migration. The antigens (like those from gut pathogens) are presented by GALT-Dendritic cells to activate naive T and B cells in inductive sites. These activated plasma cells or T-cells either home in gut effector sites by tissue-specific interactions (e.g. MadCAM-1 with $\alpha_4\beta_7$ and CCR9 with CCL25) or can move to distal sites using non tissue-specific interactions (L-selectin with PNAd and CCR5 with RANTES)
